# The readiness of addiction treatment agencies for health care reform

**DOI:** 10.1186/1747-597X-7-16

**Published:** 2012-05-02

**Authors:** Todd Molfenter, Victor A Capoccia, Michael G Boyle, Carol K Sherbeck

**Affiliations:** 1 NIATx, University of Wisconsin–Madison, 1513 University Ave, Madison, WI 53715, USA

**Keywords:** Health care reform, Addiction treatment, Substance use disorder treatment, SUD, Behavioral health, Organizational change, Care delivery, Health reform readiness index

## Abstract

The Patient Protection and Affordable Care Act (PPACA) aims to provide affordable health insurance and expanded health care coverage for some 32 million Americans. The PPACA makes provisions for using technology, evidence-based treatments, and integrated, patient-centered care to modernize the delivery of health care services. These changes are designed to ensure effectiveness, efficiency, and cost-savings within the health care system.

To gauge the addiction treatment field’s readiness for health reform, the authors developed a Health Reform Readiness Index (HRRI) survey for addiction treatment agencies. Addiction treatment administrators and providers from around the United States completed the survey located on the http://www.niatx.net website. Respondents self-assessed their agencies based on 13 conditions pertinent to health reform readiness, and received a confidential score and instant feedback.

On a scale of “Needs to Begin,” “Early Stages,” “On the Way,” and “Advanced,” the mean scores for respondents (n = 276) ranked in the Early Stages of health reform preparation for 11 of 13 conditions. Of greater concern was that organizations with budgets of < $5 million (n = 193) were less likely than those with budgets > $5 million to have information technology (patient records, patient health technology, and administrative information technology), evidence-based treatments, quality management systems, a continuum of care, or a board of directors informed about PPACA.

The findings of the HRRI indicate that the addiction field, and in particular smaller organizations, have much to do to prepare for a future environment that has greater expectations for information technology use, a credentialed workforce, accountability for patient care, and an integrated continuum of care.

## Background

On March 23, 2010, the Patient Protection and Affordable Care Act (PPACA) was signed into law, heralding imminent and significant reform for health care delivery and health insurance coverage in the United States [1]. The PPACA expands essential benefits for the insured, increases eligibility for public programs among the underserved, and offers the uninsured and underinsured greater access to affordable health insurance through state and federal health insurance exchanges [2-9]. Health reform seeks to reduce health care costs by improving prevention and wellness [10]; integrated care [11]; health information technology implementation [12-14]; evidence-based treatment [15,16]; workforce development [17]; and quality management [18] in the health care delivery system.

Within this context, we wanted to gauge the addiction treatment field’s readiness for health reform. The Health Reform Readiness Index (HRRI) survey was developed through the Accelerating Reform Initiative (ARI) conducted by NIATx, a process improvement learning collaborative at the University of Wisconsin–Madison. Through a competitive request for proposal process, ARI selected 21 community-based behavioral health organizations committed to preparing for the PPACA. In ARI, these organizations began to confront the practical implications of the PPACA requirements on care delivery and business operations. Their experiences provided the foundation for development of the HRRI survey.

The HRRI survey was designed as:

1) A self-assessment survey for addiction agencies to gauge their readiness for health reform;

2) An opportunity for addiction agencies to create awareness of what’s needed to thrive in the changing landscape of addiction treatment and health care; and

3) A method to gather information on the collective status of health reform readiness among addiction agencies in the United States.

The HRRI development process had four phases. In the first phase, a survey instrument was drafted based on the researchers’ knowledge of the PPACA and what the ARI organizations reported they needed to do in order to prepare for this legislation. The feedback mostly focused on what organizations would need in order to participate in state-based health insurance exchanges. Organizations anticipated these health insurance mechanisms would require having infrastructure for billing systems, electronic health records, outcomes tracking, and quality management, as well as being part of a continuum of care that included primary health care services. In the second phase, the ARI organizations were asked to pilot test the survey by applying the instrument to their organization. After the pilot test, organizations gave feedback on the clarity of the survey questions and how they measured intended PPACA constructs. As a result of the focus group feedback, one indicator was removed. The excluded item was related to facilities and asked participants to rate if their facilities were up to date and modern. The participants felt this indicator was too subjective and did not coincide with the intent of the PPACA. The feedback also resulted in several wording changes to the survey. During the final phase of survey development, the ARI participants, as part of their completion activities in the nine-month ARI project, applied the survey one more time and provided feedback to assure the survey’s ability to measure intended constructs. The feedback provided supported the construct validity of the revised survey and this version of the survey was used for the Health Reform Readiness Index (HRRI) assessment described in this study.

The final HRRI included two categories of reform readiness conditions: “Building Blocks,” and “Your Organization.” The Building Block conditions were 1) Consumer/Family Role, 2) Evidence-Based Treatment, 3) Accountability for Patient Care, and 4) Integrated Continuum of Care. “Your Organization” conditions were 5) Board of Directors, 6) Workforce, 7) Patient Records, 8) Holistic Care, 9) Outcomes Measurement, 10) Quality Management, 11) Health Technology, 12) Information Technology, and 13) Finance. Each condition had an indicator of health reform readiness. (Table 1)

**Table 1:** Summary of health reform readiness index categories, conditions and indicators

**CATEGORY: BUILDING BLOCKS**	
**Condition**	**Score and progression of reform readiness indicators (summary)**
Patient/Family Role	0 = Patients and family are not involved in treatment decision-making.
	1 = Patients and family are somewhat involved but clinicians make all decisions.
	2 = Patients are actively involved in treatment decision-making and goal-setting; families are invited to some sessions/events.
	3 = Patients and clinicians are full partners in treatment decision-making and goal-setting; families are involved in treatment sessions/events.
Evidence-Based Treatment	0 = Does not use National Quality Forum (NQF) practice standards.
	0 = Does not use National Quality Forum (NQF) practice standards.
	1 = Clinicians have access to prescribing medications and learning about NQF clinical interventions through training.
	2 = Has on-staff prescribing capacity. Offers in-service training for NQF clinical interventions.
	3 = On-staff prescribing capacity is widely used. Has in-service training and mechanisms for reviewing fidelity to NQF clinical interventions.
Accountability for Patient Care	0 = Documents care provided within organization over time.
	1 = Documents care provided within organization and elsewhere – information shared by patient.
	2 = Documents care provided within organization and elsewhere – information shared by patient and/or other healthcare organizations.
	3 = Documents care provided within organization and elsewhere – information shared by patient and/or other healthcare organizations). Patient identifies organization as medical home.
Integrated Continuum of Care	0 = Offers a single level of care.
	1 = Controls/has direct access to multiple levels of addiction or mental health care.
	2 = Controls/has direct access to all levels of addiction and mental health care.
	3 = Controls/has direct access to all levels of addiction, mental health, and primary care.
**CATEGORY: YOUR ORGANIZATION **	
Board of Directors	0 = Board is uninformed about parity and health care reform.
	1 = Board is informed about opportunities presented by parity and health care reform.
	2 = Board is informed and supports staff efforts to take advantage of opportunities presented by parity/reform opportunities.
	3 = Board assures all activities take advantage of opportunities presented by parity/reform in finance, operations, human resources, treatment quality, or programming
Workforce	0 = Has < 20% licensed clinicians.
	1 = Has > 20% licensed clinicians. Patients have access to medical personnel.
	2 = Has > 33% licensed clinicians and > 10% staff are medical personnel.
	3 = Has > 50% licensed clinicians and > 15% staff can prescribe medications.
Patient Record	0 = Uses only paper records.
	1 = Uses electronic records.
	2 = Uses pre-formatted electronic records which integrates into data management and billing systems.
	3 = Uses pre-formatted electronic records which integrates into data management and billing systems. Shares clinical information and patient registries electronically with other health care partners.
Holistic Care	0 = Provides only substance abuse treatment. Does not refer to other services.
	1 = Provides only substance abuse treatment, and refers patients to primary care and support services.
	2 = Provides substance abuse treatment, assesses patients’ physical and psychosocial health, and has formal agreements to refer patients to other services.
	3 = Provides substance abuse treatment, assesses patients’ physical and psychosocial health, and can transfer patients and records to other health/support organizations.
Outcomes Measurement	0 = Collects data on dates and types of service.
	1 = Collects data on dates, types of service, admissions and length of stay. Uses data for process improvement.
	2 = Collects data on dates, types of service, admissions, length of stay and patient functioning during treatment. Uses data for process improvement.
	3 = Collects data on dates, types of service, admissions, length of stay, patient functioning during treatment, and outcomes measures. Uses data for process improvement.
Quality Management	0 = Documents quality indicators. Does not have quality management staff.
	1 = Documents quality indicators. A staff person monitors requirements for licensing, payer contracts and accreditation.
	2 = Documents quality indicators. Monitors requirements for licensing, payer contracts and accreditation. Has a quality management officer and conducts regular quality reviews.
	3 = Documents quality indicators. Monitors requirements for licensing, payer contracts and accreditation. Has a quality management officer. Conducts regular quality reviews, and has a culture of continuous improvement and high level of accreditation.
Patient Health Technology	0 = Does not collect data to use in treatment.
	1 = Patients complete assessments using electronic media.
	2 = Patients complete assessments, and have access to records and clinician communication using electronic media.
	3 = Patients complete assessments, have access to records and clinician communication, and interactive support/ direction using electronic media.
Administrative Information Technology (IT)	0 = Has paper and/or electronic systems that do not interact.
	1 = IT system collects and manages utilization and financial information for billing and accounting.
	2 = IT system collects and manages utilization and financial information for billing and accounting, and links directly to billing system.
	3 = IT system collects and manages utilization and financial information. Data system is integrated for management, billing, human resources, and clinical data.
Finance	0 = Revenue mostly from grants. Does not bill third-party payers.
	1 = Up to 10% revenue comes from third-party payers. All services have unit costs.
	2 = Up to 30% revenue comes from third-party payers. All services have unit costs, and organization has cash reserves up to 90 days.
	3 = Up to 50% revenue from third-party payers. All services have unit costs, and organization has cash reserves up to 90 days.

The survey tool used a rating scale from “Needs to Begin” (score = 0) to “Advanced” (score = 3), with a progression of organizational competencies (indicators of reform readiness) as possible answers for each Condition question. Table 1 summarizes the scores and progression of organizational competencies for each Condition.

### Research questions

The study team addressed two research questions: 1) Is the addiction treatment field sufficiently prepared for the effects of PPACA?, and 2) Are organizations with budgets > $5 million more prepared for the effects of PPACA than those with smaller budgets?

### Methodology

The final HRRI survey was placed on the NIATx website [19] in October 2010 and was available to addiction treatment administrators and providers from around the United States who were interested in completing the survey. A set of health reform resources, along with a link to the HRRI survey, was included in the October 2010 NIATx eNews, an electronic newsletter. The eNews was distributed to a 4,982 member e-mail list developed from the e-mail addresses of participants of NIATx activities. In addition to the eNews announcement, those who registered for the 2011 State Associations of Addiction Services (SAAS) National Conference/NIATx Summit were encouraged to complete the online survey prior to attending the conference in July. This Summit and NIATx educational activities focus on research and practices that encourage and support the use of process improvement and organizational change techniques to improve systems of health care delivery.

The data was collected between October 1, 2010 and July 15, 2011 and was compiled using Microsoft SQL Server software. The survey contained questions about organizational characteristics (addiction services offered, number of patients served per year, annual budget, and revenue source), and 13 single-item questions on key conditions for health reform readiness. The four responses available for each condition question were: 0 = Need to Begin; 1 = Early Stages; 2 = On the Way; 3 = Advanced. The data was analyzed using Microsoft Excel and SPSS software. An analysis of the organizational characteristics of the survey participants compared to the population of NIATx participants and national data is described in the results section below.

For data analysis, the socio-demographic data are reported as percentages. The HRRI measures are reported as means. The survey results are segmented by organizations with annual budgets of < $1 million, $1-5 million, $5-10 million, and > $10 million. This study will test if significant differences of p < .01 exist for the HRRI measures due to organizational budget size. The study team used a Kendall tau-b to test the association between this pair of ordinal variables within our sample.

## Results

### Organization characteristics

#### Number of patients served

**Figure 1:**
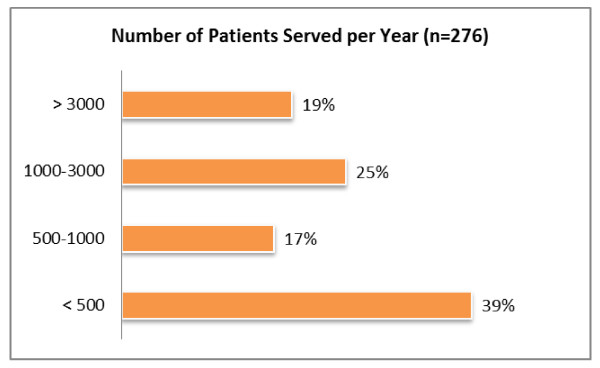
Number of patients served per year by organizations completing the HRRI survey.

More than half the 276 respondents are relatively small sites with 1,000 persons or less served per year (Figure 1). An analysis of the survey sample suggests that the survey population is similar (in terms of annual budget, types of services and number of patients served) to the population that uses NIATx services (which includes participants in grant-supported NIATx learning communities, research projects, and the annual NIATx Summit).Organizational statistics from those activities indicates 42% of participants serve fewer than 500 patients per year, 23% serve 500–1000 patients, 20% serve 1000–3000 patients, and 15% serve greater than 3000 patients. A chi-square goodness of fit analysis failed to reject the hypothesis of equal distribution and found no significant difference between the overall NIATx participant population and the sample that participated in the HRRI study (*p* = .213).

#### Substance abuse treatment services provided

89% of the 276 survey participants provide outpatient services, and 55% provide residential treatment. Less than a third of providers offer detoxification (28%) or crisis stabilization (26%). Only 15% provide vocational support for clients, and primary care services are minimal (6%). Comparable national data from the Substance Abuse and Mental Health Services Administration (SAMHSA) 2010 National Survey of Substance Abuse Treatment Services (N-SSATS) has the percentage of providers offering outpatient services at 81%, residential services at 32%, and detoxification services at 21%. As compared to national data, the HRRI sample appears to represent agencies that offer a greater number of services because the sample had a greater percentage of the following services: residential (55% study versus 32% nationally), outpatient (89% study versus 81% nationally), and detoxification (study 28% versus 21% nationally) [20].

#### Annual budget

Most of the respondents operate on a relatively small annual budget. Just over a third (39%) had an annual budget of $1-5 million, and another 31% operate on less than $1 million a year. Only 19% have an annual budget of more than $10 million.

### Conditions for health reform readiness

All but two of the 13 HRRI conditions had mean responses that scored in the “Needs to Begin” or “Early Stages” range (.5-1.49). Two of the conditions (Patient/Family Role and Quality Management) registered in the lower end of the “On the Way” range (1.5-2.49). Table 2 provides the means and standard deviations for survey respondents by total (n = 276) and by budget size.

**Table 2:** Mean scores for HRRI conditions

**Conditions**		**Total**	**<$1M** ** Budget**	**$1-5M** ** Budget**	**$5-10M** ** Budget**	**$ > 10M** ** Budget**
		**N = 276**	**N = 85**	**N = 107**	**N = 52**	**N = 32**
**Building Blocks**						
Patient/Family Role	Mean	1.57	1.61	1.50	1.58	1.69
	STD	.873	.952	.840	.871	.780
Evidence-based Treatment	Mean	1.14	.81	1.21	1.46	1.31
	STD	.911	.852	.866	.959	.896
Accountability for Patient Care	Mean	1.28	1.24	1.26	1.29	1.44
	STD	1.008	1.054	.984	1.035	.948
Integrated Continuum of Care	Mean	1.25	1.04	1.27	1.44	1.41
	STD	.798	.932	.734	.669	.712
**Your Organization**						
Board of Directors	Mean	1.08	.91	.99	1.23	1.56
	STD	1.015	.971	.957	1.113	1.014
Workforce	Mean	1.03	.93	1.05	1.13	1.09
	STD	.850	.884	.905	.687	.818
Patient Record	Mean	1.01	.93	.86	1.29	1.28
	STD	.924	.910	.936	.776	1.023
Holistic Care	Mean	1.43	1.40	1.40	1.48	1.50
	STD	.794	.805	.775	.874	.718
Outcomes Measurement	Mean	1.45	1.45	1.44	1.40	1.58
	STD	1.069	1.160	1.011	1.034	1.105
Quality Management	Mean	1.53	1.24	1.50	1.77	2.03
	STD	1.032	.972	1.076	.921	.967
Patient Health Technology	Mean	.44	.41	.40	.52	.50
	STD	.585	.583	.584	.641	.568
Admin. Information Technology (IT)	Mean	1.12	.94	1.04	1.29	1.56
	STD	.965	1.004	.910	.997	.840
Finance	Mean	1.23	1.16	1.13	1.40	1.47
	STD	.936	.962	.943	.846	.950

“Needs to Begin = 0”, “Early Stages = 1”, “On the Way =2“, or “Advanced = 3”.

#### Highest scoring conditions

The four conditions that received the highest mean scores were Patient/Family Role (1.57), Quality Management (1.53), Outcomes Measurement (1.45), and Holistic Care (1.43).

For Patient/Family Role, 88.4% of organizations indicated patients are asked about their treatment goals. 54.7% of organizations said patients understand their options for levels and types of care and are involved in selecting them. 40.6% of organizations indicated families are involved in assessment and invited to some events. An additional 14.1% indicated families also participate in treatment.

In the area of Quality Management, 81.2% indicated they have a staff member who monitors requirements for licensing, payer contracts, and accreditation; but 50.0% do not have a quality management department or officer. Conversely, 50.0% reported having a quality management officer, and regular quality reviews of clinical management and business and operating processes. Just 21.7% of organizations report having a continuous quality improvement culture and a high level of accreditation.

Outcomes Measurement also scored relatively higher with 21% of the 276 respondents reporting being “Advanced” in the area of outcomes measurement and data collection on admissions, patient functioning, patients’ substance use, employment, education, housing, and family recovery support. 26.4% indicated they were “On the Way,” collecting data on patient functioning during treatment and using that data to modify treatment plans. In contrast, 23.6% indicated they still need to begin Outcomes Measurement activities. These agencies collect basic data (dates and types of services), but do not collect additional data to assess and modify treatment plans or implement process improvement activities.

Holistic Care also scored well, given that 46% reported that they refer patients to other providers for primary care or support services; 34.8% include assessment of a patient’s physical and psychosocial health. Just 9.1% reported having the ability to transfer patients and their records to other organizations for health care and support services.

#### Lowest scoring conditions

Of the 13 readiness conditions measured in the Index, the four lowest scoring Conditions were Patient Health Technology (0.44), Patient Record (1.01), Workforce (1.03) and Board of Directors (1.08).

The addiction field is still in the early stages of implementing patient-centered health technology (lowest-scoring condition) and electronic patient records (second-lowest scoring condition) in the clinical setting. In terms of leveraging health technology to engage and enhance client treatment and recovery, it’s use is limited. 40.2% of the respondents did have electronic assessment tools available to their patients. However, just 2.5% provided patients electronic access to their records, test results and clinician communication.

In the area of Patient Record, 38.8% of survey participants indicated they still use only paper records. 61.2% did have some form of electronic health record and 33.3% of the survey sample indicated those records are integrated with into data management and billing systems. While a mere 3.3% who have electronic records actually share clinical information and patient registries electronically with other health partners.

The third lowest-scoring condition was Workforce: 27.5% indicated that less than 20% of their organization’s clinicians are licensed, and they do not have access to medical personnel who can prescribe medications as part of addiction treatment. Just under half of the 276 respondents (48.6%) indicated that 20-33% of their clinician FTEs or staff hours are licensed. Only 6.9% scored “Advanced,” indicating that more than half of their clinician or staff hours are licensed and more than 15% of staff can prescribe medications.

The role of Board Members also scored lower than other conditions: 39.9% of respondents reported that their board does not know much about health care reform and parity. Only 8% indicated that their board assures that all the organization’s activities take advantage of opportunities presented by parity and health reform legislation in the areas of finance, operations, human resources, treatment quality, or programming.

The Kendall tau-b analysis compared organizations with different annual budgets. Larger budget size was related to greater use of evidence-based treatment, a continuum of care, electronic patient record, quality management, and administrative information systems (at *p* < .01) (Table 3). Once the data was segmented by organizations with less than or greater than $5 million budgets, organizations with greater than $5 million in annual revenue had average scores better than those with less than $5 million in all variables (Table 3). When the Kendall tau-b analysis of budget size compared budgets greater and less than $5 million, one additional variable became significant: Board of Directors, suggesting that organizations with a budget over $5M are more likely to have Boards of Directors who are more informed about health reform and parity.

**Table 3:** Comparison of organizational budget size and health reform readiness* (** = ***p*** **< .01)**

**Variables**	**All Budget Sizes (<$1M, $1-5M, $5-10M, $10 + M) Approx. Sig.**	**<$5M Budget v. ≥$5M Budget Approx. Sig.**
BUILDING BLOCKS		
Patient/Family Role	.905	.610
Evidence-based Treatment	.000**	.002**
Accountability for Patient Care	.519	.455
Integrated Continuum of Care	.000**	.001**
YOUR ORGANIZATION		
Board of Directors	.012	.004**
Workforce	.045	.092
Patient Record	.008**	.000**
Holistic Care	.710	.586
Outcomes Measurement	.972	.863
Quality Management	.000**	.000**
Patient Health Technology	.248	.157
Administrative Information Technology (IT)	.003**	.001**
Finance	.062	.013

* Kendall tau-b test of association.

## Conclusion and discussion

The HRRI scores suggest that addiction treatment organizations are in the early stages of preparing for changes anticipated with the PPACA. Accordingly, most organizations will have to address many areas in order to prepare for the PPACA and to be successful in the future. Organizational size does affect readiness for PPACA. Larger organizations scored better for all conditions. Smaller organizations are going to have to make significant changes to patient records, evidence-based treatment application, quality management functions, and become part of the health care continuum of care. To achieve these goals, they may need to look more aggressively for partnerships and collaborations, and pursue specific actions to prepare for health reform.

Specifically, organizations need to implement electronic health records that can efficiently report quality data and bill health insurers for services. Once implemented, organizations should strive to achieve electronic connectivity with other providers in their community hopefully using the inter-operability standards developed by the area’s Community Health Information Network (CHIN).

For evidence-based treatments, the HRRI emphasized the use of medication-assisted therapy (MAT). Organizations needed to have, at a minimum, MAT referral capacity in order to gain a score of “On the Way” or higher in the HRRI. Pharmacotherapy is a standard approach in general medicine and it was projected by HRRI developers that MAT capacity would be a necessary competency needed to participate in health networks.

Managed care organizations, accountable care organizations, and other health insurers prefer to work with health networks that provide a continuum of care because they can more efficiently deliver a comprehensive set of health services. This approach has been the case in general medicine as health insurance plans develop contractual relationships with networks of hospitals and primary care providers. Addiction treatment organizations that can demonstrate they are part of existing health networks can more easily be integrated into the provider panels of health insurance plans. These networks should be able to provide multiple levels of addiction and mental health services as well as provide primary care access.

Managed care organizations also typically seek evidence that an organization has a quality management function. This is often a necessary function and, when in place, can sometimes allow treatment agencies to request a higher fee for their services. Addiction treatment organizations should have a quality management function that conducts regular quality reviews of clinical management and business and operating processes. This function should be able to quantify the quality of services provided, explain how poor performance can be identified, and provide examples of how quality performance has been improved.

The study design had limitations that should be taken into consideration when interpreting the results. Although the results are based on a large sample of organizations (n = 276), the results were derived from a convenience sample of visitors to the NIATx website and a set of conference attendees. Of the 276 HRRI respondents, 20.7% were conference attendees, representing 54 organizations. Conference attendees have discretionary resources that may not be present for non-attendees. Both the conference and the NIATx website had a process improvement focus and may attract organizations that demonstrate more organizational flexibility to conduct organizational change. Those who completed the survey on the NIATx website could be considered to be motivated to learn more about PPACA and how it might impact their organization. Moreover, the sample represented organizations that tended to have greater variety of services than organizations nationally due to greater provision of outpatient, residential, and detoxification services. Overall, survey participants may have been more apt to have resources available for conference attendance, a process improvement focus, an interest in the ramifications of PPACA, and offer a greater variety of services to clients.

While these factors limit the study, they also provide an interesting perspective. If many of the HRRI respondents are indeed motivated to improve their delivery of care, offer a greater variety of services, and are better resourced to prepare for health reform, but still lag behind in terms of reform readiness (as our results suggest), then we may infer that less motivated addiction treatment organizations are even further behind in preparation for changes the PPACA brings when fully implemented in 2014. Educating and equipping unprepared organizations is imperative if they are to compete and flourish in a changing environment that requires patient-centered services, evidence-based treatment, accountability for outcomes, effective use of health information technologies, and fiscal agility to provide better addiction treatment. Organizations with smaller budgets may have greater needs to prepare for reform. Hence, many smaller substance abuse treatment agencies will need to prepare for a broad set of organizational changes or consider partnering with other organizations to access greater resources.

A recurring refrain in the field is that addiction agencies are reacting slowly to the potential changes by the PPACA due to doubt that the legislation will be fully enacted. This may not be a prudent strategy because many of the changes called for by the PPACA are consistent with macro-environmental changes already in process for addiction services, such as use of managed care and electronic health records. Failure to adequately prepare could give other health care providers the opportunity to offer addiction services instead. In sum, the PPACA attempts to make provisions for modernizing the delivery of health care services. The HRRI provides a stark reminder to the addiction field that historic changes to service delivery and structures are anticipated, and that the field must adapt and keep pace in order to survive and thrive in this new environment.

## Abbreviations

ARI, Accelerating Reform Initiative; HRRI, Health Reform Readiness Index; N-SSATS, National Survey of Substance Abuse Treatment Services; NIATx, Network for the Improvement of Addiction Treatment, University of Wisconsin–Madison; PPACA, Patient Protection and Affordable Care Act; SAAS, State Associations of Addiction Services; SAMHSA, Substance Abuse and Mental Health Services Administration.

## Competing interests

The authors declare that they have no competing interests.

## Author’s contributions

TM, VC and MB designed the study. TM and CS collected and analyzed the data. All authors participated in drafting the manuscript. All authors read and approved the final manuscript.

## Authors’ information

TM is an assistant scientist at the University of Wisconsin–Madison College of Engineering and a deputy director in the Center for Health Enhancement System Studies (CHESS). He has been a principal investigator and deputy director on multiple grants and has spent the last 20 years studying, planning, and leading organizational and individual change efforts. VC is a senior scientist at the University of Wisconsin–Madison College of Engineering’s Center for Health Enhancement System Studies. He has been working for the last 10 years with foundations and government on programs to improve access to and quality of addiction and mental health services. MB is associate researcher at the University of Wisconsin–Madison College of Engineering’s Center for Health Enhancement System Studies. He is the former CEO of Fayette Companies, a behavioral health treatment organization located in Peoria, Illinois. CS is an outreach specialist at the University of Wisconsin–Madison College of Engineering’s Center for Health Enhancement System Studies.
